# The role of gender in the perception of autism symptom severity and future behavioral development

**DOI:** 10.1186/s13229-019-0266-4

**Published:** 2019-03-29

**Authors:** Philippine Geelhand, Philippe Bernard, Olivier Klein, Bob van Tiel, Mikhail Kissine

**Affiliations:** 1 ACTE at LaDisco and ULB Neuroscience Institute, Université libre de Bruxelles, CP 175, 50 avenue F.D. Roosevelt, 1050 Brussels, Belgium; 2 CeSCUP, Université libre de Bruxelles, CP122, 50 avenue F.D. Roosevelt, 1050 Brussels, Belgium; 3 Leibniz-Zentrum Allgemeine Sprachwissenschaft (ZAS), Schützenstr. 18, 10117 Berlin, Germany

**Keywords:** Sex ratio, Gender bias, Concern, Caregiver, Adolescence, Symptom severity

## Abstract

**Background:**

Increasing attention is being paid to the higher prevalence of boys with Autism Spectrum Disorder (ASD) and to the implications of this ratio discrepancy on our understanding of autism in girls. One recent avenue of research has focused on caregiver’s concern, suggesting that autism might present differently in boys and girls. One unexplored factor related to concerns on child development is whether socio-cultural factors such as gender-related expectations influence the evaluation of symptom severity and predictions about future behavioral development.

**Methods:**

The latter concerns were the focus of the present study and were explored by investigating laypeople’s judgment of the severity of autism symptoms using an online parent role-playing paradigm, in which participants were asked to rate vignettes depicting the behaviors of a child in different everyday life scenarios. The child’s gender and the severity of ASD symptoms were manipulated to examine the effect of gender on the perception of symptom severity.

**Results:**

Results suggest that there are no gender differences in perceived symptom severity and associated degree of concern for 5-year-old boys and girls but that there is a gender difference in perceived future atypicality at 15 years old, with boys being rated as more likely to be perceived as atypical by their peers at that age than girls.

**Conclusions:**

Investigating parent’s cognition about their child’s future behavioral development can provide additional information regarding delayed diagnosis of autistic girls.

## Background

An imbalanced sex ratio already featured in Kanner’s [1] and Asperger’s [2] descriptions of autism and has proven a robust characteristic of research samples ever since then, with the most recent estimates suggesting a ratio of three males for one female diagnosed with autism [3]. Research on autism spectrum disorder (ASD) is thus grounded in male-dominant samples. As a consequence, the way we conceptualize, measure, and diagnose autism probably revolves around a male-centric presentation [4]. A male-biased understanding of autism has dramatic implications for autistic girls—perhaps even more so for those without cognitive impairments, as this sub-group tends to be diagnosed less frequently and later in life (e.g., [5]). Meanwhile, early identification and treatment of developmental disorders like ASD is crucial for optimizing outcomes [6, 7].

Previous research strongly suggests that autism is more difficult to detect in girls (e.g., [4, 8]), but the underlying reasons for this difficulty remain unclear. A promising approach to address this question is to explore caregivers’ concerns (e.g., [4, 9]). In the vast majority of cases, caregivers are key in drawing professional attention to their child’s development. Overall, studies on the reliability of first-hand concerns suggest that parent observations can predict autism diagnosis [10, 11]. A few studies have also investigated whether potential differences in caregivers’ concern for autistic boys and girls could impact the path to diagnosis. For example, [4] found that externalizing behaviors, such as hitting or yelling in a social setting, was reported as their primary concern by half of the caregivers of autistic girls, but only by a quarter of caregivers of autistic boys. By contrast, internalizing behaviors (e.g., withdrawal) were more commonly reported as a concern for boys.

Such findings highlight the potential influence of social expectations on caregivers’ perception of child behavior. For example, research on typical populations suggests that shyness is more tolerated in girls than in boys, while, conversely, aggression is socially more acceptable in boys than in girls (e.g., [12, 13]). Hence, because passivity and compliance are traits typically attributed to and expected of girls, excessive shyness may be a greater cause for concern when displayed by a boy than by a girl; consequently, internalizing or withdrawal behaviors are more likely not to raise concern in a girl. In autism research, population-based studies have suggested that at comparable levels of severity of autistic traits, females are less likely than males to obtain an autism diagnosis [8, 14], further supporting the existence of greater concern for boys than girls. Together, these findings suggest the possibility that caregiver (and professional) attention to and perception of behavior severity may be influenced by the child’s gender.

A child’s peers may also play a crucial role in how this child’s parents perceive her. Behaviors deviating from the norm, such as important social withdrawal (viz. withdrawing from social activities and peer groups), is associated with peer rejection [15]. Studies on typical boys and girls who are considered as solitary-anxious suggest that solitary-anxious boys are more likely to be rejected by their peers and experience emotional difficulties than solitary-anxious girls (e.g., [16, 17]). For example, [16] found that although high anxious-solitude (i.e., withdrawal due to shyness and social anxiety) predicted higher exclusion trajectories both for boys and for girls, the exclusion trajectory was significantly greater for anxious-solitary boys than anxious-solitary girls, suggesting that anxious-solitary boys face a higher risk of interpersonal difficulties than anxious-solitary girls. This gender difference is rather consistent across studies and has been attributed to the fact that solitary-anxious boys violate gender norms of male confidence and self-assuredness [16, 17]. Interestingly, if children demonstrating solitary-anxious behaviors also demonstrated attention-seeking or aggression, gender differences disappeared, with boys and girls equally likely to be rejected by peers. Since peer rejection is likely to attract the attention of teachers and parents and raise their concerns, this latter finding may partly explain why autistic girls are more likely to obtain a timely diagnosis if they display conspicuous difficulties such as aggressive behavior [5].

Furthermore, peer rejection during childhood predicts negative outcomes during the adolescent and early adulthood periods (e.g., [18]). For instance, peer-rejected children are more likely to develop both externalizing and internalizing problems than their better-accepted counterparts. Hence, because peer rejection leads to later behavioral maladjustment and because boys are more likely to be rejected for displaying gender atypical behavior, parents might be more concerned for their son’s future if he displays atypical behavior in childhood than for their daughter. This assumption is consistent with findings that girls are believed to be more likely to naturally “grow out” of atypical behaviors, in contrast to boys (e.g., [19, 20]).

Another important, albeit seldom discussed point, is that gender-biased expectations tend to change with age. In the transition to adolescence, girls are confronted with more complex social expectations than boys, putting higher pressure on them to be socially acute (e.g., [21]). The existence of increased social expectations for female teenagers is supported by findings on adolescent’s interests and preferences, which suggest that girls spend more times in relationship-related activities, while boys spend more time alone, playing ball or video games (e.g., [22]). Furthermore, girls’ same-sex friendships involve greater intimacy and caring than boys’ same-sex friendships, which are more characterized by friendly rivalry and risky activities (e.g., [22, 23]). In line with these expectations, we speculate that reaching adolescence, girls become more likely to experience harsh social sanctions when displaying atypical behaviors such as social withdrawal than during childhood as these will now contrast with the gender- and age-specific expectations of more sophisticated social skills.

Adolescence is a difficult period for typical girls, and even more so for autistic girls, who must deal with both the difficulties inherent in adolescence and those associated with their diagnosis. For example, a recent study found that girls demonstrate fewer problems with social communication than boys early on, but that their skills aggravate by adolescence [24]. These findings seem to coincide, again, with the observation that autistic girls receive a later diagnosis more often than boys do [5]. It remains unclear, at this stage, whether social difficulties have a later onset in autistic girls or whether pre-existing, milder difficulties are brought out due to the increasing complexity of these girls’ social environment during adolescence [24].

Since gender stereotypes can influence social perception [23] and, furthermore, are likely to evolve with age, it is important to understand how gender may influence our perception of atypical behavior displayed by a child, as well as our expectations about this child’s later development. The aim of our study was to directly examine whether perceived severity of behavior—and hence magnitude of concern—could vary as a function of gender, as well as of an interaction between gender and age. To gain insight into the role of gender stereotypes on behavior perception and future behavioral development, we investigated laypeople’s judgment of the severity of autism symptoms. We used an online parent role-playing paradigm, in which participants were asked to rate the behaviors of a “fictitious” child. There are two main reasons for using this method. First, gender-related expectations are socio-cultural products and, as such, are predicted to spread to virtually each of society’s members; the choice of layman participants is thus likely to reflect parent’s attitudes at the early pre-diagnosis stage. Second, role-playing circumvents some of the methodological limitations and confounds present in previous studies, such as small sample sizes, different levels of cognitive abilities of children [25], and influence of diagnostic process on retrospective reports (e.g., [26]).

Participants were recruited online and asked to put themselves in the shoes of a parent of a 5-year-old child. They were then presented with a rating questionnaire involving items depicting different everyday situations in which “their” child displayed various types of behaviors. Participants were asked to read these descriptions and rate the severity of their child’s behavior, likelihood to seek expert advice, and future atypicality of the child (at 15 years old^1^). Our predictions were twofold: first, we predicted that gender would influence the perception of the child’s current behavior, with girls’ behavior being rated as less worrisome and less likely to prompt expert advice. Second, regarding the child’s future prognosis at the age of 15 years, we expected participants to predict that boys would be perceived by their peers as more atypical than girls.

## Methods

### Participants

Participants were recruited on the online crowdsourcing platform Prolific. To access the questionnaire, participants had to meet the following criteria: (1) be US citizens and (2) be native English speakers^2^ and (3) not having participated in the study pretests.

Four hundred participants took part in the test questionnaire.^3^ Participants were excluded if they did not fully complete the questionnaire or if they failed the attention or the gender checks.^4^ This led to the removal of 15 participants (3.8%). The final sample included 385 participants (192 males), mean age = 32.69 years old (SD = 11.12, age range = 18–64).

### Material and design

The algorithm items of the Autism Diagnostic Interview-Revised (ADI-R, [27]), a standard diagnostic tool of autism, were used as basis to create concrete, real-life examples of the relevant behavior in a 5-year-old child. Each item came in three degrees of severity: severe, moderate, and typical. Some items of the algorithm were not included as they either referred to behaviors present at or before 36 months^5^ or because it was difficult to create a natural-sounding typical example of the behavior at hand,^6^ as they tend not to occur in typically developing children. We ended up with a final selection of 24 items. See Table 1 for an example item.

**Table 1:** Example of an item in each experimental condition (severe, moderate, and typical)

Condition	Item: offer of comfort
Severe	The other day, you baby-sat your 4-year-old nephew. While going to the kitchen, he bumped his head on the door and started to cry. Your child was also going to the kitchen but did not stop or look at him and passed him to go to the kitchen.
Moderate	The other day, you baby-sat your 4-year-old nephew. While going to the kitchen, he bumped his head on the door and started to cry. Your child who saw the whole scene, went to sit next to him but did not do or say anything.
Typical	The other day, you baby-sat your 4-year-old nephew. While going to the kitchen, he bumped his head on the door and started to cry. Your child who saw the whole scene, looked concerned and immediately went to sit next to him and hugged him, telling him it would be okay.

Considering the novelty of the study paradigm, the items were first pretested (without mentioning the child’s genders, as shown in Table 1) to validate the manipulations of symptom severity before assessing any potential influence of the child’s sex on the evaluations of autism-related behavior. The pretest results confirmed the manipulation of severity: severe items were rated as significantly more worrying than moderate items, which were rated as significantly more worrying than the typical items. From the initial pretest items, those for which the ratings clearly differed between the three different degrees of severity were then selected^7^ to create the test questionnaire. This resulted in seven items. Detailed results of these pretests are available online on the Open Science Framework.^8^

The seven items were modified to become “sex-specific,” viz. behaviors were described as displayed either by a 5-year-old boy or 5-year old girl. Previous studies have shown that labelling a child as a boy or a girl is sufficient to induce differential behavior (e.g., different interaction styles; [28]), and we assumed that simply mentioning the child’s sex was sufficient to activate gender-related expectations. To reflect children’s real-life behavior as accurately as possible and to avoid an order effect, questionnaire items included profiles of mixed symptoms, i.e., severe-moderate and moderate-typical. In the severe-moderate questionnaire, three of the items were severe and four were moderate. For each of the seven items to appear both as the severe version and moderate version, the profile mixing severe and moderate symptoms came in two versions (e.g., in one version, item 1 was severe, in the other version item 1 was moderate). The same was done for the mixed profile moderate-typical. A homogeneous typical profile was used as control condition. The combination of two severe-moderate, two moderate-typical, and one typical profiles with gender, female vs. male, and conditions resulted in ten conditions.^9^ Participants were assigned to only one of the ten conditions. See Table 2 for a summary of the ten conditions.

**Table 2:** Summary of the experimental conditions

Conditions	Sex	Severity
1	Female	Severe-moderate V1
2		Severe-moderate V2
3		Moderate-typical V1
4		Moderate-typical V2
5		Typical
6	Male	Severe-moderate V1
7		Severe-moderate V2
8		Moderate-typical V1
9		Moderate-typical V2
10		Typical

### Procedure

Eligible participants accessed one of the ten conditions via a link to the questionnaire on Ibex (Internet Based EXperiments). The role-playing instructions asked participants to put themselves in the shoes of a parent of a 5-year-old boy or girl while they read descriptions of seven different situations, one by one, describing everyday situations involving them and their daughter/son. Participants could read at their own pace, clicking on a link to the next item when they were ready to proceed. After reading all the descriptions, they were asked to rate as a whole:

- How worrisome they find their child’s behavior described in the situations on a scale from 1 (not at all worrisome) to 7 (extremely worrisome).
- How likely the behaviors would prompt them to seek professional advice on a scale from 1 (not at all likely) to 7 (extremely likely).
- How atypical this child would be perceived by his/her peers at 15-years-old on scale from 1 (typical) to 7 (atypical).

To examine whether parental status (viz. having children or not) and knowledge of autism (viz. recognizing autism in the descriptions) could influence participants ratings, we asked participants the following questions after they had provided their ratings of children’s behavior:

- Do you have children of your own?
- If you have children, do any of them have a mental or physical disability?
- After reading all these situations, did a disorder come to mind?
- If yes, which one?

### Statistical analysis

The effects of symptom severity and child’s gender on participants’ ratings of worry towards the described behavior, likelihood to seek professional advice, and atypicality were analyzed with cumulative link models in R [29] using the *clm* function from the *ordinal* package [30]. All categorical variables were dummy-coded. Post hoc comparisons of least-squares means, with Tukey adjustment for multiple comparisons were performed using the *lsmeans* package [31].

As our participants came from a crowdsourcing platform, we controlled for factors that could influence their ratings, namely participants’ characteristics^10^(age, gender, and SES), parental status,^11^and knowledge of autism.^12^ None of these factors influenced the significance of the effects on the ratings and were therefore not included in the models.

## Results

### Ratings of worry and likelihood to seek professional advice

Cumulative link models with the additive effects of severity and gender revealed a significant effect of severity on ratings of worry (*χ*^*2*^ (2) = 320.46, *p* < 0.0001) and likelihood to seek professional advice (*χ*^*2*^ (2) = 259.631, *p* < 0.0001), but no significant effect of gender (*χ*^*2*^ (1) = 1.05, *p* = 0.3; *χ*^*2*^ (1) = 0.1, *p* = 0.7, respectively). See Tables 3 and 4.

**Table 3:** Cumulative link model with additive effects of symptom severity and gender

Ratings of worry towards described behavior	Estimate	Standard error
Moderate-typical^a^	− 2.86	(0.25)***
Typical	− 5.73	(0.39)***
Male^a^	0.19	(0.19)
Number of observations	385	

Significance codes: 0 “***”, 0.1 “ ”

^a^Severe-moderate symptoms and gender female are the reference levels

**Table 4:** Cumulative link model with additive effects of symptom severity and gender

Ratings of likelihood to seek professional advice	Estimate	Standard error
Moderate-typical^a^	− 2.49	(0.24)***
Typical	− 4.89	(0.36)***
Male^a^	0.07	(0.19)
Number of observations	385	

Significance codes: 0 “***”, 0.1 “ ”

^a^Severe-moderate symptoms and gender female are the reference levels

Post hoc comparisons indicated that both for boys and girls, items in the *severe-moderate* condition received higher ratings than those in the *moderate-typical* (*z* = − 11.226, *p* < 0.0001) and *typical* conditions (*z* = − 14.663, *p* < 0.0001) and those in the *moderate-typical* condition received higher ratings than those in the *typical* condition (*z* = − 8.586, *p* < 0.0001). See Table 5.

**Table 5:** Mean ratings of worry per condition

	*N*	Severe-moderate M (SD)	*N*	Moderate-typical M (SD)	*N*	Typical M (SD)
Male	77	5.43 (1.19)	78	3.12 (1.48)	36	1.53 (0.97)
Female	78	5.27 (1.32)	77	2.97 (1.33)	39	1.46 (1.21)

Post hoc comparisons on ratings of likelihood to seek expert advice indicate that for both boys and girls, items in the *severe-moderate* condition received higher ratings than those in the *moderate-typical* (*z* = − 10.385, *p* < 0.0001) and *typical* conditions (*z* = − 13.406, *p* < 0.0001) and those in the *moderate-typical* condition received higher ratings than those in the *typical* condition (*z* = − 7.493, *p* < 0.0001). See Table 6.

**Table 6:** Mean ratings likelihood (per condition)

	*N*	Severe-moderate M (SD)	*N*	Moderate-typical M (SD)	*N*	Typical M (SD)
Male	77	5.56 (1.46)	78	2.82 (1.72)	36	1.58 (1.18)
Female	78	5.30 (1.77)	77	3.02 (1.65)	39	1.41 (1.21)

### Ratings of atypicality

There was a significant effect of severity (*χ*^*2*^ (2) = 97.182, *p* < 0.0001) and gender (*χ*^*2*^ (1) = 4.095, *p* = 0.04) on ratings of perceived atypicality by peers at 15 years old. Participants in the *severe-moderate* conditions rated the child at 15 years old as more atypical by their peers than participants did in the *moderate-typical* and *typical* conditions, and boys received higher ratings of perceived future atypicality than girls. See Table 7.

**Table 7:** Cumulative link model with additive effects of symptom severity and gender (severe-moderate symptoms and gender female are the reference levels, standard errors are between brackets)

Ratings of perceived atypicality by peers at 15 years old	Estimate	Standard error
Moderate-typical^a^	− 1.17	(0.21)***
Typical	− 2.69	(0.29)***
Male^a^	0.37	(0.18)*
Number of observations	385	

Significance codes: 0 “***”, 0.01 “*”

^a^Severe-moderate symptoms and gender female are the reference levels

Post hoc comparisons indicated children in the *severe-moderate* condition received higher ratings of atypicality than those in the *moderate-typical* (*z* = − 5.636, *p* < 0.0001) and *typical* conditions (*z* = − 9.291, *p* < 0.0001), and children in the *moderate-typical* condition received higher ratings of atypicality than those in the *typical* condition (*z* = − 5.619, *p* < 0.0001). See Table 8.

**Table 8:** Mean ratings for perceived atypicality by peers at 15 years old (per condition)

	*N*	Severe-moderate M (SD)	*N*	Moderate-typical M (SD)	*N*	Typical M (SD)
Male	77	5.56 (1.46)	78	4.64 (1.6)	36	3.0 (1.94)
Female	78	5.23 (1.65)	77	4.13 (1.52)	39	2.95 (1.88)

There was no significant interaction between severity and gender on the ratings of atypicality (*χ*^*2*^ (2) = 0.86, *p* = 0.65), indicating that independently of symptom severity, boys received higher ratings of perceived atypicality at 15 years old than girls.

## Discussion

In the present study, a novel paradigm involving perspective-taking of a parent to rate the behaviors of a 5-year-old child was used to explore whether the child’s gender would modulate the perceived severity of symptomatic behaviors. This was achieved by measuring the magnitude of worry towards the behaviors, subsequent likelihood to seek professional advice, and future atypicality. Our manipulations of symptom severity led to significant differences in ratings of worry across conditions, suggesting that participants considered more severe behaviors as more worrying than moderate symptoms and typical behaviors. Moreover, more severe behaviors were also rated as more likely to prompt participants to seek professional advice.

However, contrary to our initial assumptions, child’s gender did not yield any significant differences in the ratings of worry and likelihood to seek medical advice. One possible explanation for this lack of effect is that parental concerns towards behaviors were investigated with explicit measures, viz. that the ratings of the behaviors required deliberate evaluation on the part of the participants. These results do not rule out a dissociation between explicit and implicit attitudes (e.g., [32]). Specifically, the explicit beliefs or stances that we take do not always align with the implicit associations we hold. As has been shown in previous studies on implicit race and gender biases (e.g., [33–35]), it is possible that in our experiment, the child’s gender did not have any influence on deliberate processing, such as explicit ratings of behaviors, but could surface in more implicit measures of judgments.

Another possibility, raised by a reviewer, is that participants might have been more engaged with reading the vignettes and understanding the behavioral descriptions than paying attention to the child’s gender.

Our results about peer-perceived future atypicality provide initial support for the possibility of surfacing in more implicit measures of judgments. Indeed, despite similar ratings of symptom severity for boys and girls, participants predicted boys to be peer-perceived as more atypical at the age of 15 years than girls. This suggests that gender stereotypes did influence the perception of atypical behaviors and that gender differences surfaced in the assumptions made about the evolution of these behaviors and future social adjustment of boys and girls. This explanation aligns with previous findings that girls are believed to be more likely to naturally “grow out” of atypical behaviors, in contrast to boys (e.g., [19, 20]).

One way to explore this assumption could be to examine the causes parents attribute to their child’s behavior and whether these vary as a function of their child’s gender. Although we could not explore this hypothesis directly in the current study, we believe parent cognition provide a promising avenue, as previous studies have shown that parental responses to behaviors are mediated by parent cognition (rather than being directly elicited by child behaviors, e.g., [36]). Therefore, the causal explanations parents make about their child’s behavior are likely to influence both their immediate behavioral responses toward the child and their general choice of parenting strategies [37, 38]. Particularly relevant for the present discussion are studies on causal attribution that found that parents make gender-differentiated attributions (among others [39–42]). Our results are also in line with studies suggesting that judgments of severity and typicality are, to a certain degree, independent of one another [43]. What is considered (a) typical is subject to social and cultural norms; for this reason, evaluations of typicality are more likely to be influenced by social stereotypes and biases than ratings of severity and concerns.

Parents’ response to their child’s behavior may be influenced by the nature of the cause they attribute to that behavior—which might itself be influenced by the child’s gender, perhaps even more than by the magnitude or even the presence of concerns. Future research should focus on the direction of the relationship between factors such as child’s gender, type of causal attribution and parents’ response, and how such relationships would relate particularly to the evaluation of behaviors and symptoms of autism.

## Limitations

One obvious limitation of our study is that questionnaire items were all based on items used in the ADI-R diagnostic algorithm, which includes only the core symptoms of ASD. Some studies suggest that while there are few significant quantitative sex differences in the core symptoms (e.g., [44]), differences between the female and male presentations do surface when comparing associated characteristics of ASD [25, 44]. A possible avenue for future research on potential gender differences could be to include autism-related behaviors and/or associated comorbidities.

## Conclusion

The results of this study highlight important future research directions on sex ratio in autism spectrum disorder (ASD). The contribution of socio-cultural factors (e.g., gender-based expectations) and type of cognitions (e.g., causal attributions) to the unequal gender ratio in ASD should not be underestimated. Gaining further insight into such factors is crucial; as to date, the first step to an ASD diagnosis essentially relies on caregivers’ perception of the child’s behavior, and the diagnostic assessment itself relies on behavioral descriptions. Understanding the relationships and interactions between gender expectancies, types of cognitions and responses to behaviors should be beneficial for the diagnostic process and tools designed to ascertain a diagnosis and, in the longer term, to the design of treatment interventions.

## Footnotes

1 Psychologists divide the developmental period of adolescence in three subperiods, early adolescence (10–14 years), middle adolescence (14–18 years), and late adolescence (18–22 years). We thought that the age 15 (middle adolescence) would represent a prototypical adolescent, making it easier for participants to imagine their fictitious child at that age.

2 As most of the literature on gender in autism, including epidemiological data, is based on the population from the USA, we decided to pool our participants from the same population.

3 Sample size for questionnaire 3 was determined on basis of an a priori statistical power analysis in the software G*Power 3.1.

4 Attention check was operationalized as a question for which participants had to indicate the rating “3” and gender check by asking participants whether they had seen the profile of a 5-year-old boy, 15-year-old boy, 5-year-old girl, or 15-year-old girl.

5 All items from section D: *Atypical development at or before 36 months* (item 2, item 9, item 10, item 86, and item 87).

6 Item 33 (stereotypical utterances and delayed echolalia), item 37 (pronominal inversion), item 38 (neologisms/idiosyncratic language), item 39 (verbal rituals), item 65 (friendship), item 67 (unusual preoccupation), item 70 (rituals/compulsion), item 71 (unusual sensory interests), item 78 (other complex mannerisms or stereotypical body movements), and item 79 (hand movements in the middle).

7 Item 34 (social verbalization), item 35 (reciprocal conversation), item 36 (inappropriate comments/questions), item 50 (direct gaze), item 55 (offer of comfort), item 57 (diversity of facial expressions to communicate), and item 69 (repetitive use of objects or interest for parts of objects)

8 Link to pretest material: https://osf.io/ewt7d/?view_only=f53d2ccb2c3f494aa0741a34b3e98f94

9 Link to full questionnaires: https://osf.io/ewt7d/?view_only=f53d2ccb2c3f494aa0741a34b3e98f94

10 SES was a participant characteristic provided by Prolific. For 79 participants, SES was not available.

11 Full analyses are available on Open Science Framework: https://osf.io/ewt7d/?view_only=f53d2ccb2c3f494aa0741a34b3e98f94

12 Full analyses are available on Open Science Framework: https://osf.io/ewt7d/?view_only=f53d2ccb2c3f494aa0741a34b3e98f94
